# A randomized, open-label, phase 3 trial of pembrolizumab plus epacadostat versus sunitinib or pazopanib as first-line treatment for metastatic renal cell carcinoma (KEYNOTE-679/ECHO-302)

**DOI:** 10.1186/s12885-023-10971-7

**Published:** 2024-07-25

**Authors:** Primo N. Lara, Luis Villanueva, Carolina Ibanez, Mustafa Erman, Jae Lyun Lee, Daniel Heinrich, Oleg Nikolaevich Lipatov, Craig Gedye, Erhan Gokmen, Alejandro Acevedo, Andrey Semenov, Se Hoon Park, Rustem Airatovich Gafanov, Fatih Kose, Mark Jones, Xiaoqi Du, Mihaela Munteanu, Rodolfo Perini, Toni K. Choueiri, Robert J. Motzer

**Affiliations:** 1 University of California Davis Comprehensive Cancer Center, University of California Davis, 4501 X Street, Davis, Sacramento, CA 95817 USA; 2 Oncology Department, Instituto Oncologico Fundacion Arturo Lopez Perez, Santiago, Chile; 3 Hematology and Oncology Department, Pontificia Universidad Católica de Chile, Santiago, Chile; 4 Department of Medical Oncology, Hacettepe University Medical Faculty, Ankara, Turkey; 5 Department of Oncology and Internal Medicine Asan Medical Center, University of Ulsan College of Medicine, Ulsan, South Korea; 6 Department of Oncology, Akershus University Hospital, Lørenskog, Norway; 7 Department of Oncology and Radiotherapy, Innlandet Hospital Gjøvik, Gjøvik, Norway; 8 Department of Oncology, Republican Clinical Oncology Dispensary, Ufa, Russia; 9 Department of Medical Oncology, Calvary Mater Newcastle, Waratah, NSW Australia; 10 Faculty of Medicine, Ege University, Izmir, Turkey; 11 ONCOCENTRO APYS Clinical Research Unit, Viña del Mar, Chile; 12 Ivanovo Regional Oncology Dispensary, Ivanovo, Russia; 13 Department of Hematology and Oncology, Samsung Medical Center, Sungkyunkwan University School of Medicine, Seoul, South Korea; 14 Russian Scientific Center of Roentgenoradiolog, Moscow, Russia; 15 Department of Medical Oncology, Baskent University, Ankara, Turkey; 16 Incyte Corp, Wilmington, DE USA; 17 Merck & Co., Inc, Rahway, NJ USA; 18 Dana-Farber Cancer Institute, Boston, MA USA; 19 Genitourinary Oncology Service, Department of Medicine, Memorial Sloan Kettering Cancer Center, New York, NY USA

**Keywords:** Renal cell carcinoma, Programmed death 1, PD-1, Indoleamine 2,3-deoxygenase 1, IDO1, Pembrolizumab, Epacadostat

## Abstract

**Background:**

Immunotherapy-based combinations have emerged as standard therapies for patients with metastatic renal cell carcinoma (mRCC). Pembrolizumab, a PD-1 inhibitor, combined with epacadostat, an indoleamine 2,3-deoxygenase 1 selective inhibitor, demonstrated promising antitumor activity in a phase 1 study in advanced solid tumors, including mRCC.

**Methods:**

KEYNOTE-679/ECHO-302 was a randomized, open-label, parallel-group, multicenter, phase 3 study (NCT03260894) that compared pembrolizumab plus epacadostat with sunitinib or pazopanib as first-line treatment for mRCC. Eligible patients had histologically confirmed locally advanced or metastatic clear cell RCC and had not received systemic therapy. Patients were randomly assigned 1:1 to pembrolizumab 200 mg IV every 3 weeks plus epacadostat 100 mg orally twice daily versus sunitinib 50 mg orally once daily (4 weeks on treatment followed by 2 weeks off treatment) or pazopanib 800 mg orally once daily. Original dual primary end points were progression-free survival and overall survival. Enrollment was stopped when a phase 3 study in melanoma of pembrolizumab plus epacadostat compared with pembrolizumab monotherapy did not meet its primary end point. This protocol was amended, and primary end point was changed to investigator-assessed objective response rate (ORR) per RECIST 1.1.

**Results:**

One-hundred-twenty-nine patients were randomly assigned to receive pembrolizumab plus epacadostat (*n* = 64) or sunitinib/pazopanib (*n* = 65). Median (range) follow-up, defined as time from randomization to data cutoff, was 10.3 months (2.2–14.3) and 10.3 months (2.7–13.8) in the pembrolizumab plus epacadostat and sunitinib/pazopanib arms, respectively. ORRs were similar between pembrolizumab plus epacadostat (31.3% [95% CI 20.2–44.1] and sunitinib/pazopanib (29.2% [18.6–41.8]). Grade 3–5 treatment-related adverse events occurred in 34.4% and 42.9% of patients in the pembrolizumab plus epacadostat and sunitinib/pazopanib arms, respectively. One patient in the sunitinib/pazopanib arm died of septic shock (not treatment-related). Circulating kynurenine levels decreased in the pembrolizumab plus epacadostat arm, but not to levels observed in healthy subjects.

**Conclusions:**

ORRs were similar between pembrolizumab plus epacadostat and sunitinib/pazopanib as first-line treatment in patients with mRCC. Safety and tolerability appeared similar between treatment arms; no new safety concerns were identified. Antitumor responses observed in patients with RCC receiving pembrolizumab plus epacadostat may be driven primarily by pembrolizumab.

**Clinical trial registration:**

ClinicalTrials.gov; NCT03260894.

**Supplementary Information:**

The online version contains supplementary material available at 10.1186/s12885-023-10971-7.

## Background

Cancer of the kidney and renal pelvis was estimated to be diagnosed in 76,080 people in the United States in 2021, with 13,780 projected to die of the disease [1]. The most common histologic subtype is clear cell renal cell carcinoma (RCC) [2, 3]. For patients with relapsed or de novo stage IV disease, the preferred first-line treatment options include immune checkpoint inhibitor–based therapies such as axitinib plus pembrolizumab, axitinib plus avelumab, lenvatinib plus pembrolizumab, cabozantinib plus nivolumab or ipilimumab plus nivolumab [2, 4–8]. Specifically, in patients with disease considered intermediate or poor risk per the International Metastatic RCC Database Consortium (IMDC), options include all 5 immunotherapy-based doublets and cabozantinib monotherapy, whereas for all IMDC risk categories, axitinib plus pembrolizumab, cabozantinib plus nivolumab, or pembrolizumab plus lenvatinib have emerged as preferred standard regimens [2, 4–8]. In selected patients not eligible for immunotherapy, treatment with a vascular endothelial growth factor receptor (VEGFR) tyrosine kinase inhibitor (eg, sunitinib, pazopanib, cabozantinib) is often offered [2].

Because of toxicities and disease progression with currently available therapies, there is an unmet medical need for safer and more effective combination treatments for metastatic RCC (mRCC). Both indoleamine 2,3-deoxygenase 1 (IDO1) and programmed death 1 (PD-1) mediated pathways suppress T-cell–mediated antitumor immunity, and IDO1 and the PD-1 ligand (PD-L1) are co-expressed in multiple cancer types and correlate with poor prognosis [9–15]. Anti–PD-1 agents block immunosuppressive receptor PD-1 on T cells, thus enhancing immune responses against tumors [16]. IDO is an enzyme that catalyzes the rate-limiting step in the conversion of tryptophan to kynurenine, which results in apoptosis of effector T cells and activation of regulatory T cells, thereby promoting an immunosuppressive environment and tumor growth [17]. Further, high expression of IDO1 may be associated with resistance to PD-1 inhibition in non–small cell lung cancer, melanoma, and RCC [18–21]. Combining an anti–PD-1 agent with an IDO1 inhibitor may therefore enhance antitumor immunity. In preclinical models, anti–PD-1 treatment combined with an IDO1 inhibitor showed synergistic antitumor activity in models of melanoma and glioblastoma [22, 23].

Pembrolizumab is a potent and highly selective humanized monoclonal antibody directed against PD-1 that has shown promising antitumor activity as monotherapy in both advanced clear cell RCC and non-clear cell mRCC and is approved, in combination with axitinib [4, 24, 25] or with lenvatinib [26, 27], for the first-line treatment of advanced mRCC. Epacadostat (formerly INCB024360) represents a novel, potent, and selective inhibitor of IDO1 in human tumor and dendritic cells [20, 28, 29]. In a phase 1 study of patients with advanced solid tumors, the combination of pembrolizumab plus epacadostat has shown promising antitumor activity, including objective responses in patients with treatment-naive and previously treated melanoma, non–small cell lung cancer, urothelial cancer, and mRCC (25 of 62 patients responded, including 2 of 11 with RCC) [30]. These responses were durable across tumor types, with 68% of responses (17 of 25) ongoing at data cutoff.

The aim of the present study was to compare the antitumor activity and tolerability of pembrolizumab plus epacadostat with standard of care treatment (at the time of study conduct) with either sunitinib or pazopanib as first-line therapy in patients with mRCC.

## Methods

This was a randomized, open-label, parallel-group, multicenter, phase 3 trial (KEYNOTE-679/ECHO-302; NCT03260894; first registration: 8/24/2017). The study was conducted at 74 centers in 14 countries in accordance with the Declaration of Helsinki and Good Clinical Practice guidelines, and independent institutional review boards or ethics committees reviewed and approved the protocol and applicable amendments for each institution (Supplemental Table 1). All patients provided written informed consent.

### Patients

Eligible patients had histologically confirmed locally advanced or mRCC (stage IV per American Joint Committee on Cancer) with a clear cell component, with or without sarcomatoid features, no prior systemic therapy for mRCC, measurable disease per RECIST 1.1, and Karnofsky performance status (KPS) ≥ 70. Patients were excluded if they had previously received therapy with an anti–PD-1, anti–PD-L1, or anti–PD-L2 agent, or with epacadostat or any anti-IDO1 agent, or with an agent directed to another stimulatory or co-inhibitory T-cell receptor. Patients were ineligible if they had previously received therapy with VEGF/VEGFR or with mammalian target of rapamycin–targeting agents for locally advanced or metastatic cancer or if they received systemic anti-cancer therapy, including investigational agents, within 4 weeks before randomization.

### Treatments

Patients were randomly assigned 1:1 to receive pembrolizumab 200 mg IV every 3 weeks plus epacadostat 100 mg orally twice daily continuously or sunitinib or pazopanib (sunitinib 50 mg orally once daily [6-week cycles; 4 weeks on treatment followed by 2 weeks off treatment] or pazopanib 800 mg orally once daily continuously). Dose delays or dose reductions were performed per defined criteria in the protocol. If pembrolizumab dosing was held, dosing for epacadostat was also to be held. Patients who required dose reduction of epacadostat because of adverse events (AEs) remained at the lower dose; re-escalation was not permitted.

Randomization was performed centrally using an interactive voice response system/integrated web response system and stratified according to IMDC risk category (favorable vs intermediate vs poor), physician’s intended choice of comparator drug (sunitinib vs pazopanib), and geographical region (United States, Canada, and Western Europe vs rest of world).

### Study conduct

The study was initiated on December 7, 2017, and on May 2, 2018, the sponsor made a strategic decision to stop enrollment permanently because a phase 3 study in patients with unresectable or metastatic melanoma did not meet the prespecified primary end point of improvement in progression-free survival (PFS) for pembrolizumab plus epacadostat compared with pembrolizumab plus placebo [31]. In that study, no new safety concerns had arisen with pembrolizumab plus epacadostat compared with pembrolizumab monotherapy. In the present study, patients who experienced ongoing clinical benefit could continue study treatment at the discretion of the investigator and would continue to be monitored.

### End points

The original dual primary end points were comparison of PFS (as assessed by blinded, independent central review) and overall survival (OS) between treatment arms. The protocol was amended when enrollment was stopped, and the primary end point changed to estimation of objective response rate (ORR; as assessed per RECIST v1.1 by the investigator) for each treatment arm after the first on-study scan (week 12); PFS and OS were removed as end points.

ORR was defined as the proportion of patients in the analysis population whose best response was complete response (CR) or partial response (PR). Protocol-specified efficacy imaging was stopped at week 12 with the protocol amendment. Subsequent disease monitoring was performed according to local standard of care. For some patients imaging was completed beyond week 12 at the time enrollment was stopped; thus, ORR was based on all available imaging assessments.

Safety and tolerability were assessed as a secondary end point and included the number of patients with an AE and the number of patients who discontinued because of an AE.

Pharmacodynamic activity, an exploratory end point, was assessed as change from baseline to week 3 in serum kynurenine in both treatment arms. Serum kynurenine levels were determined using a proprietary, validated liquid chromatography–mass spectrometry assay using calibrated standards (Worldwide Clinical Trials, Morrisville, NC). Kynurenine serum levels at cycle 1, day 1 (C1D1) and cycle 2, day 1 (C2D1) were compared using paired *t*-tests within each treatment arm.

### Statistical analysis

The original target enrollment was 630 patients; a subsequent amendment (June 15, 2018) resulted in enrollment being stopped early after 129 patients had been randomly assigned. In the original statistical analysis plan, PFS and OS, dual original primary end points, were to be evaluated using a stratified log-rank test, and hazard ratio was to be estimated using a stratified Cox regression model. Event rates over time were to be estimated within each treatment group using the Kaplan–Meier method. In the amended protocol, ORR was estimated within each treatment arm with 95% confidence intervals (CI) using Clopper-Pearson exact method based on binomial distribution [32] and was summarized by study treatment group. Counts and percentages of patients with AEs were provided by treatment group. AEs were assessed using Common Terminology Criteria for Adverse Events version 4.0.

The intention-to-treat population was used for the efficacy analysis and included all randomly assigned patients. The all-patients-as-treated population was used for the safety analysis and included all randomly assigned patients who received at least 1 dose of study treatment. Safety and tolerability parameters were summarized by descriptive statistics based on treatment group.

## Results

### Patients

**Fig. 1:**
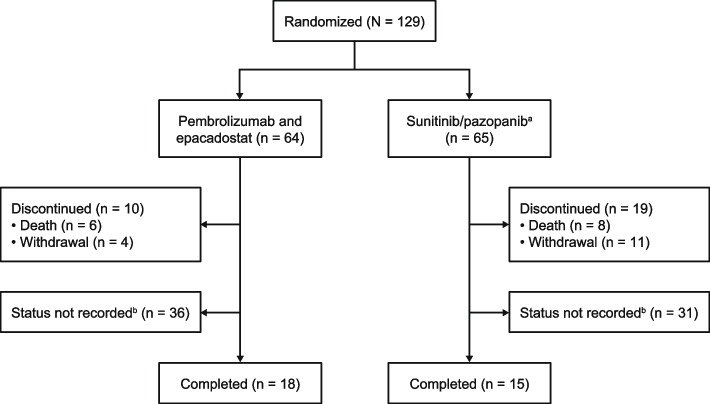
Patient disposition for trial. ^a^Sunitinib 50 mg orally once daily (6-week cycles; 4 weeks on treatment followed by 2 weeks off treatment) or pazopanib 800 mg orally once daily continuously. ^b^Status was not reported as of the data cutoff date. Patients could be ongoing with study or treatment

The study was initiated on December 7, 2017, and enrollment was stopped on May 2, 2018. Median (range) follow-up, defined as time from randomization to data cutoff, was 10.3 months (2.2–14.3) in the pembrolizumab plus epacadostat arm and 10.3 months (2.7–13.8) in the sunitinib/pazopanib arm. A total of 129 patients were randomly assigned to treatment (pembrolizumab plus epacadostat, *n* = 64; sunitinib/pazopanib, *n* = 65); 10 patients discontinued in the pembrolizumab plus epacadostat arm and 19 patients discontinued in the sunitinib/pazopanib arm (11 primarily because of withdrawal) (Fig. 1). The primary reason for discontinuation in the pembrolizumab plus epacadostat arm was death (*n* = 6); none of these deaths were deemed treatment related. Treatment arms were balanced with regard to most patient characteristics (Table 1). Overall, 42.6% were aged ≥ 65 years, and most patients were male (72.9%), white (84.5%), had Eastern Cooperative Oncology Group performance status 0 (58.9%) or 1 (39.5%). Metastatic staging at initial diagnosis was M0 (44.2%) or M1 (55.0%). IMDC risk category was favorable in 29.5% of patients, intermediate in 55.0%, and poor in 15.5% at time of study entry.

**Table 1:** Patient demographics

	**Pembrolizumab + Epacadostat** ***n* = 64**	**Sunitinib or Pazopanib** ***n* = 65**	**Total** ***N* = 129**
Sex,* n* (%)			
Male	44 (68.8)	50 (76.9)	94 (72.9)
Female	20 (31.2)	15 (23.1)	35 (27.1)
Age,* n* (%)			
< 65 years	35 (54.7)	39 (60.0)	74 (57.4)
≥ 65 years	29 (45.3)	26 (40.0)	55 (42.6)
Mean (SD)	62.9 (10.9)	62.1 (10.6)	62.5 (10.7)
Race,* n* (%)			
American Indian or Alaska native	1 (1.6)	0 (0)	1 (0.8)
Asian	10 (15.6)	9 (13.9)	19 (14.7)
White	53 (82.8)	56 (86.2)	109 (84.5)
Ethnicity,* n* (%)			
Hispanic/Latino	19 (29.7)	14 (21.5)	33 (25.6)
Not Hispanic / Latino	43 (67.2)	47 (72.3)	90 (69.8)
Not reported	2 (3.1)	4 (6.2)	6 (4.7)
ECOG performance status,* n* (%)			
0	41 (64.1)	35 (53.9)	76 (58.9)
1	23 (35.9)	28 (43.1)	51 (39.5)
2	0 (0)	2 (3.1)	2 (1.6)
Karnofsky Performance Scale,* n* (%)			
100	32 (50.0)	25 (38.5)	57 (44.2)
80–90	29 (45.3)	34 (52.3)	63 (48.8)
70	3 (4.7)	6 (9.2)	9 (7.0)
Geographic region,* n* (%)			
US	5 (7.8)	9 (13.9)	14 (10.9)
Non-US	59 (92.2)	56 (86.2)	115 (89.2)
Metastatic staging at initial diagnosis,* n* (%)			
M0	28 (43.8)	29 (44.6)	57 (44.2)
M1	36 (56.3)	35 (53.8)	71 (55.0)
Missing	0 (0)	1 (1.5)	1 (0.8)
Brain metastases,* n* (%)			
Yes	1 (1.6)	0 (0)	1 (0.8)
No	63 (98.4)	65 (100.0)	128 (99.2)
IMDC risk category,* n* (%)			
Favorable	19 (29.7)	19 (29.2)	38 (29.5)
Intermediate	36 (56.3)	35 (53.9)	71 (55.0)
Poor	9 (14.1)	11 (16.9)	20 (15.5)
Sarcomatoid histology,* n* (%)			
Yes	4 (6.3)	6 (9.2)	10 (7.8)
No	36 (56.3)	35 (53.9)	71 (55.0)
Unknown	24 (37.5)	22 (33.9)	46 (35.7)
Missing	0 (0)	2 (3.1)	2 (1.6)

*Abbreviations*: *ECOG* Eastern Cooperative Oncology Group, *IMDC* International Metastatic RCC Database Consortium

### Response rates

Response rates were similar in both treatment arms (Table 2). Objective responses were achieved by 20 patients (31.3% [95% CI 20.2–44.1]) receiving pembrolizumab plus epacadostat and by 19 patients (29.2% [95% CI 18.6–41.8]) receiving sunitinib/pazopanib. One patient, enrolled in the pembrolizumab plus epacadostat arm, achieved CR. The disease control rate (CR + PR + stable disease) was similar in both treatment arms (45 patients [70.3%] in the pembrolizumab plus epacadostat arm vs 46 [70.8%] in the sunitinib/pazopanib arm).

**Table 2:** Objective response rate per RECIST v1.1. by investigator assessment^a^

	**Pembrolizumab + Epacadostat** ***n* = 64**		**Sunitinib or Pazopanib** ***n* = 65**	
	***n* (%)**	**95% CI**^**b**^	***n* (%)**	**95% CI**^**b**^
CR	1 (1.6)	0.0–8.4	0 (0)	0.0–5.5
PR	19 (29.7)	18.9–42.4	19 (29.2)	18.6–41.8
Objective response rate (CR + PR)	20 (31.3)	20.2–44.1	19 (29.2)	18.6–41.8
Stable disease	25 (39.1)	27.1–52.1	27 (41.5)	29.4–54.4
Disease control rate (CR + PR + SD)	45 (70.3)	57.6–81.1	46 (70.8)	58.2–81.4
Progressive disease	17 (26.6)	16.3–39.1	12 (18.5)	9.9–30.0
Not evaluable	0 (0)	0.0–5.6	0 (0)	0.0–5.5
No assessment	2 (3.1)	0.4–10.8	7 (10.8)	4.4–20.9
ORR per IMDC risk category				
Favorable	4/19 (21.1)	6.1–45.6	6/19 (31.6)	12.6–56.6
Intermediate/poor	16/45 (35.6)	21.9–51.2	13/46 (28.3)	16.0–43.5
Intermediate	14/36 (38.9)	23.1–56.5	10/35 (28.6)	14.6–46.3
Poor	2/9 (22.2)	2.8–60.0	3/11 (27.3)	6.0–61.0

*Abbreviations*: *CR* complete response, *ECOG* Eastern Cooperative Oncology Group, *IMDC* International Metastatic RCC Database Consortium, *PR* partial response, *RECIST* Response Evaluation Criteria in Solid Tumors, *SD* stable disease

^a^Responses are based on investigator assessments per RECIST v1.1 without confirmation using all scans up to the cutoff date of February 28, 2019

^b^95% CI on objective response based on Clopper-Pearson (exact) method

**Fig. 2:**
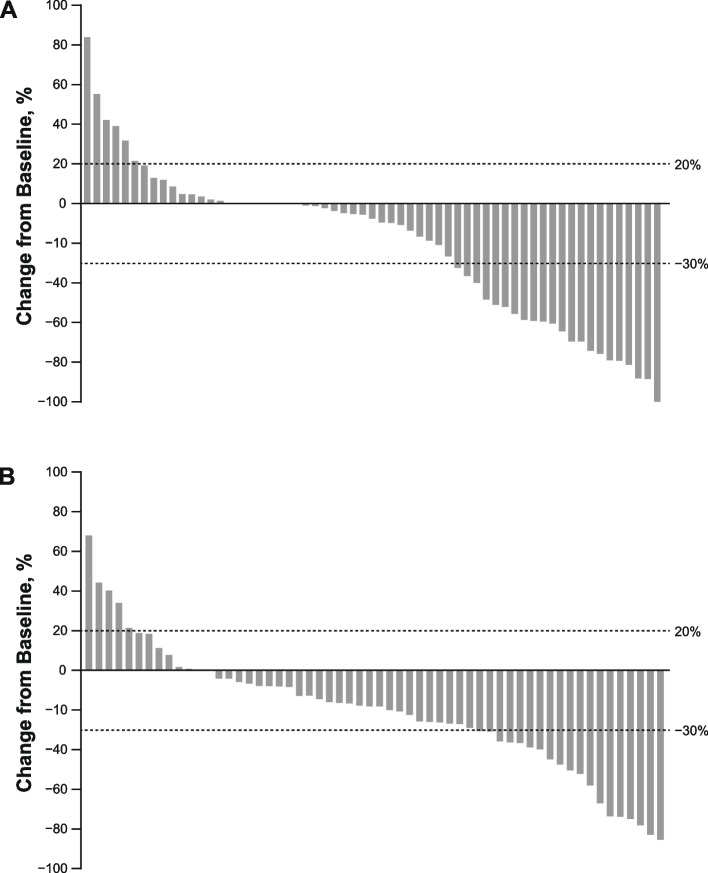
Best tumor change from baseline in patients with measurable disease at baseline and at least 1 postbaseline measurement in the (**a**) pembrolizumab plus epacadostat arm or the (**b**) sunitinib or pazopanib arm. ^a^Based on investigator assessment per RECIST v1.1 in the ITT population which included all subjects with measurable disease at baseline and ≥ 1 post-baseline measurement by cutoff date. Abbreviations: *ITT* intention-to-treat, *RECIST* Response Evaluation Criteria in Solid Tumors

Change in tumor burden also indicated a similar profile of tumor response in both treatment arms; 62% of patients receiving pembrolizumab plus epacadostat and 78% of those receiving sunitinib/pazopanib therapy experienced a reduction in the sum of the diameter of target lesions (Fig. 2).

In patients with favorable IMDC risk category, the ORR was 21.1% (95% CI 6.1–45.6) with pembrolizumab plus epacadostat versus 31.6% (95% CI 12.6–56.6) with sunitinib/pazopanib. In patients with intermediate or poor IMDC risk category, the ORR was 35.6% (95% CI 21.9–51.2) with pembrolizumab plus epacadostat versus 28.3% (95% CI 16.0–43.5) with sunitinib/pazopanib.

### Safety

At least 1 AE occurred in all patients in both the pembrolizumab plus epacadostat arm and the sunitinib/pazopanib arm (Table 3). Overall, 81.3% of patients in the pembrolizumab plus epacadostat arm and 93.7% of patients in the sunitinib/pazopanib arm had AEs that were considered by the investigator to be related to study drug. Grade 1/2 treatment-related AEs occurred in 65.6% versus 57.1% of patients, and grade 3–5 treatment-related AEs occurred in 34.4% versus 42.9% of patients in the pembrolizumab plus epacadostat arm versus the sunitinib/pazopanib arm. There were no treatment-related deaths in either treatment arm; 1 patient in the sunitinib/pazopanib arm died of septic shock (not treatment related (per AMA)). All-cause AEs occurring in ≥ 10% of patients in the pembrolizumab plus epacadostat arm and the sunitinib/pazopanib arm were nausea (34.4% vs 31.7%), pruritus (25.0% vs 1.6%), diarrhea (20.3% vs 47.6%), and fatigue (18.8% vs 23.8%) (Table 4). Eighteen patients (28.1%) in the pembrolizumab plus epacadostat arm and 15 patients (23.8%) in the sunitinib/pazopanib arm had serious AEs.

**Table 3:** Summary of adverse events^a^

	**Pembrolizumab + Epacadostat** ***n* = 64**	**Sunitinib or Pazopanib** ***n* = 63**
Any AE	64 (100.0)	63 (100.0)
Grade 3–5 AE^b^	34 (53.1)	39 (61.9)
Any treatment-related AE^c^	52 (81.3)	59 (93.7)
Grade 3–5 treatment-related AE^b,c^	22 (34.4)	27 (42.9)
Serious AE	18 (28.1)	15 (23.8)
Serious treatment-related AE^c^	4 (6.3)	6 (9.5)
Discontinued any drug because of a treatment-related AE^c^	8 (12.5)	5 (7.9)
Discontinued pembrolizumab	5 (7.8)	0 (0)
Discontinued epacadostat	8 (12.5)	0 (0)
Discontinued sunitinib	0 (0)	4 (6.3)
Discontinued pazopanib	0 (0)	1 (1.6)
Death due to AE	0 (0)	1 (1.6)

*Abbreviations*: *AE* adverse event

^a^Nonserious and serious AEs up to 90 days after last dose are included

^b^Grades are based on national cancer institute common terminology criteria for adverse events

^c^Determined by the investigator to be related to study drug

**Table 4:** Incidence of any-grade nonserious adverse events (incidence ≥ 10% in either treatment group) regardless of attribution

Adverse event, *n* (%)	Pembrolizumab + Epacadostat *n* = 64	Sunitinib or Pazopanib *n* = 63
Nausea	22 (34.4)	20 (31.7)
Pruritus	16 (25.0)	1 (1.6)
Diarrhea	13 (20.3)	30 (47.6)
Rash	11 (17.2)	5 (7.9)
Fatigue	12 (18.8)	15 (23.8)
Anemia	10 (15.6)	12 (19.0)
Back pain	10 (15.6)	9 (14.3)
Decreased appetite	10 (15.6)	12 (19.0)
Constipation	9 (14.1)	3 (4.8)
Hypothyroidism	9 (14.1)	12 (19.0)
ALT increased	8 (12.5)	14 (22.2)
Amylase increased	8 (12.5)	9 (14.3)
Blood creatinine increased	7 (10.9)	7 (11.1)
Cough	7 (10.9)	6 (9.5)
Hypertension	7 (10.9)	23 (36.5)
Weight decreased	7 (10.9)	2 (3.2)
Blood alkaline phosphatase increased	6 (9.4)	9 (14.3)
Lipase increased	6 (9.4)	9 (14.3)
Vomiting	6 (9.4)	12 (19.0)
AST increased	4 (6.3)	16 (25.4)
Blood bilirubin increased	1 (1.6)	9 (14.3)
Dysgeusia	1 (1.6)	8 (12.7)
Headache	3 (4.7)	9 (14.3)
Palmar–plantar erythrodysesthesia	1 (1.6)	14 (22.2)
Hair color changes	0 (0)	7 (11.1)

*Abbreviations*: *ALT* alanine transaminase, *AST* aspartate transaminase

Eight patients (12.5%) in the pembrolizumab plus epacadostat arm discontinued because of a treatment-related AE; 5 patients (7.8%) discontinued pembrolizumab and 8 patients (12.5%) discontinued epacadostat. In the sunitinib/pazopanib arm, 5 patients (7.9%) discontinued because of a treatment-related AE (4 [6.3%] sunitinib and 1 [1.6%] pazopanib).

### Pharmacodynamic endpoint

Samples were available for pharmacodynamic analysis in 61 and 52 patients from the pembrolizumab plus epacadostat and the sunitinib/pazopanib arms, respectively. Serum kynurenine levels from baseline to week 3 decreased by 10.3% with pembrolizumab plus epacadostat (from 2.9 µM at baseline to 2.6 µM at week 3; absolute change, –0.3 µM) but not to levels previously reported in healthy volunteers (1.5 µM) [28] (Supplemental Fig. 1). In contrast, serum kynurenine increased by 3.2% from baseline to week 3 with sunitinib/pazopanib (from 3.1 µM to 3.2 µM; absolute change, + 3.2%).

## Discussion

Despite advances in the treatment of patients with clear cell mRCC, many patients experience disease relapse or progression. Results from the ECHO-202/KEYNOTE-037 study indicated that pembrolizumab combined with epacadostat was well tolerated and showed evidence of durable objective responses in 2 patients with mRCC, which warranted further investigation and informed the design of the current study [30]. However, the ECHO-301/KEYNOTE-252 study found that epacadostat in combination with pembrolizumab did not improve clinical benefit compared with pembrolizumab monotherapy for unresectable or metastatic melanoma [31]. Based on those findings, a decision was made to stop the current study and to change the primary end point to investigator-assessed ORR per RECIST v1.1.

In the current study, response rates were similar in patients with locally advanced or mRCC receiving first-line pembrolizumab plus epacadostat compared with response rates in those receiving treatment with sunitinib or pazopanib. Sunitinib or pazopanib were used as comparators because these were the standard treatments at the time the study was conducted. Response rates reported in this study were similar to those observed in KEYNOTE-427 with single-agent pembrolizumab in 110 patients who had previously untreated advanced clear cell RCC [24]. This suggests that the addition of epacadostat to pembrolizumab has little effect on response rates in RCC. Although interpretation of the findings of this study are limited by its relatively small sample size, response rates were numerically higher in patients with an intermediate or a poor IMDC risk category receiving pembrolizumab plus epacadostat versus sunitinib/pazopanib; conversely, response rates were lower in patients with a favorable IMDC risk category receiving pembrolizumab plus epacadostat versus sunitinib/pazopanib. Despite the small subgroups of patients, this observation is consistent with earlier observations and suggest greater benefit of VEGF-targeted therapies in favorable risk [4, 5].

Immunotherapy combinations are effective in the treatment of RCC. Pembrolizumab in combination with axitinib [4, 33] or lenvatinib [26, 27] are approved first-line treatment options for patients with locally advanced RCC or mRCC. Pembrolizumab in combination with lenvatinib also showed improved progression-free survival, overall survival, and objective response rate over sunitinib in the phase 3 CLEAR study of patients with advanced RCC [7]. Further, IDO1 expression may be a valuable biomarker for response to immune therapy in patients with mRCC. High IDO-1 expression in tumor endothelial cells in patients with mRCC is associated with better therapeutic response to nivolumab [10].

Epacadostat and other IDO-1 inhibitors, including indoximod and navoximod, are also in clinical development for other tumor types [17]. Several phase 3 studies with linrodostat (BMS-986205), a selective IDO-1 inhibitor, in combination with nivolumab for the treatment of muscle-invasive bladder cancer (NCT03661320) [34] and previously untreated metastatic or unresectable melanoma (NCT03329846) are ongoing.

Consistent with the results of the ECHO-301/KEYNOTE-252 study, pembrolizumab in combination with epacadostat was generally well tolerated [31]. AEs and AE rates were generally similar between the pembrolizumab plus epacadostat and sunitinib/pazopanib treatment arms and were consistent with data from previous studies evaluating this combination in other tumor types [31]. The pembrolizumab plus epacadostat combination was manageable, and no new safety concerns were identified. Study treatment discontinuations because of treatment-related AEs were similar in both arms. Moreover, the pharmacodynamic data showed decreases in serum kynurenine that did not reach those of healthy subjects [28, 35], suggesting that higher doses of epacadostat may be warranted. The observation that serum kynurenine levels decreased in the pembrolizumab plus epacadostat arm and slightly increased in the sunitinib/pazopanib arm is consistent with an inhibitory effect of epacadostat on IDO1 activity. Because IDO1 is induced by interferon-γ [36], it is possible that the antitumor response creates an environment in which IDO1 expression surpasses the ability of epacadostat 100 mg twice daily to sufficiently inhibit kynurenine production. In fact, a retrospective pooled analysis of epacadostat clinical studies determined that when combined with an anti-PD-1 agent, doses < 600 mg twice daily were not able to maintain inhibition of plasma kynurenine, suggesting that doses ≥ 600 mg twice daily warrant additional studies in combination with checkpoint inhibitors to determine any clinical benefit to normalizing kynurenine levels [35].

### Limitations

Given the early termination of this study, the sample size was relatively small and the duration of follow-up was short, which has to be considered when interpreting the results.

## Conclusion

Pembrolizumab plus epacadostat demonstrated similar response rates compared with sunitinib or pazopanib in patients with locally advanced RCC or mRCC who had not previously received systemic therapy. AE rates were generally similar between treatment arms and no new safety concerns were identified for either drug. When taking into account the initial promising activity of single-agent pembrolizumab in advanced RCC (KEYNOTE-427) [24, 25] and the lack of additional benefit with pembrolizumab plus epacadostat over pembrolizumab monotherapy in the melanoma setting (ECHO-301/KEYNOTE-252) [31], the findings of the current study provide further evidence that the antitumor responses observed in patients with RCC receiving pembrolizumab plus epacadostat 100 mg twice daily may be driven primarily by pembrolizumab.

### Supplementary Information

**Additional file 1: Supplemental Table 1.** List of investigators and institutional review boards.** Supplemental Figure 1.** Box and whisker plot showing change from baseline to week 3 in serum kynurenine among patients in the both the pembrolizumab plus epacadostat arm and the sunitinib/pazopanib arm. Kynurenine levels at C1D1 and C2D1 were compared using paired *t*-tests within each treatment arm The dotted line indicates median kynurenine levels in healthy subjects (1.5 μM) [1] *Epa* , epacadostat, *Pembro* pembrolizumab, *SoC* standard of care.

## Data Availability

Merck Sharp & Dohme LLC, a subsidiary of Merck & Co., Inc., Rahway, NJ, USA (MSD), is committed to providing qualified scientific researchers access to anonymized data and clinical study reports from the company’s clinical trials for the purpose of conducting legitimate scientific research. MSD is also obligated to protect the rights and privacy of trial participants and, as such, has a procedure in place for evaluating and fulfilling requests for sharing company clinical trial data with qualified external scientific researchers. The MSD data sharing website (available at: http://engagezone.msd.com/ds_documentation.php) outlines the process and requirements for submitting a data request. Applications will be promptly assessed for completeness and policy compliance. Feasible requests will be reviewed by a committee of MSD subject matter experts to assess the scientific validity of the request and the qualifications of the requestors. In line with data privacy legislation, submitters of approved requests must enter into a standard data-sharing agreement with MSD before data access is granted. Data will be made available for request after product approval in the US and EU or after product development is discontinued. There are circumstances that may prevent MSD from sharing requested data, including country or region-specific regulations. If the request is declined, it will be communicated to the investigator. Access to genetic or exploratory biomarker data requires a detailed, hypothesis-driven statistical analysis plan that is collaboratively developed by the requestor and MSD subject matter experts; after approval of the statistical analysis plan and execution of a data-sharing agreement, MSD will either perform the proposed analyses and share the results with the requestor or will construct biomarker covariates and add them to a file with clinical data that is uploaded to an analysis portal so that the requestor can perform the proposed analyses.

## Abbreviations

AE: Adverse event
ALT: Alanine transaminase
AST: Aspartate transaminase
CI: Confidence interval
CR: Complete response
ECOG: Eastern Cooperative Oncology Group
IDO1: Indoleamine 2,3-deoxygenase 1
IMDC: International Metastatic RCC Database Consortium
ITT: intention-to-treat
KPS: Karnofsky Performance Scale
mRCC: Metastatic renal cell carcinoma
ORR: Objective response rate
PD-1: Programmed death 1
PD-L1: Programmed death ligand 1
PFS: Progression-free survival
PR: Partial response
RCC: Renal cell carcinoma
RECIST: Response Evaluation Criteria in Solid Tumors
SD: Stable disease
VEGFR: Vascular endothelial growth factor receptor
