# Supplementary material for: A randomized, open-label, phase 3 trial of pembrolizumab plus epacadostat versus sunitinib or pazopanib as first-line treatment for metastatic renal cell carcinoma (KEYNOTE-679/ECHO-302)
Source: BMC Cancer. 2024 Jul 25;23(Suppl 1):1253. doi: 10.1186/s12885-023-10971-7 (PMC11270760; doi:10.1186/s12885-023-10971-7)
Supplement: Supplementary file 1 — Additional file 1: Supplemental Table 1. List of investigators and institutional review boards. Supplemental Figure 1. Box and whisker plot showing change from baseline to week 3 in serum kynurenine among patients in the both the pembrolizumab plus epacadostat arm and the sunitinib/pazopanib arm. Kynurenine levels at C1D1 and C2D1 were compared using paired t-tests within each treatment arm The dotted line indicates median kynurenine levels in healthy subjects (1.5 μM) [1] Epa , epacadostat, Pembro pembrolizumab, SoC standard of care. [file 12885_2023_10971_MOESM1_ESM.docx]

**Supplemental Table 1. List of investigators and institutional review boards**

| **Principal Investigator** | **Affiliation** | **Institutional Review Board** |
| --- | --- | --- |
| Louis Lacombe | CHU de Quebec-Universite Laval-Hotel Dieu de Quebec, Quebec, QC Canada | Medical Biomedical Research Ethics Committee, Montreal, Canada |
| Georg Bjarnason | Sunnybrook Health Sciences, Odette Cancer Centre, Toronto, ON, Canada | OCREB - Ontario Cancer Research Ethics Board, Toronto, Canada |
| Andrew Robinson | Kingston Health Sciences Centre, Kingston ON, Canada | OCREB – Ontario Cancer Research Ethics Board, Toronto, Canada |
| Jean Sebastien Aucoin | CIUSSS de la Mauricie et du Centre du Quebec, Trois-Rivieres, ON, Canada | Medical Biomedical Research Ethics Committee, Montreal, Canada |
| Daniel Heng | Tom Baker Cancer Centre, Calgary, AB, Canada | Health Research Ethics Board of Alberta – HREBA, Edmonton Canada |
| Wilson H Miller | Jewish General Hospital, Montreal, QC Canada | Medical Biomedical Research Ethics Committee, Montreal, Canada |
| Alan Berg | Southeast Nebraska Hematology & Oncology Consultants, P.C., Lincoln NE, USA | Copernicus Group Independent Review Board, Cary, NC, USA |
| Daniel Vaena | The West Clinic, P.C., Germantown, TN, USA | Western IRB, Puyallup, WA, USA |
| Stephan DiSean | Kendall  Utah Cancer Specialists, Salt Lake City, UT, USA | Copernicus Group Independent Review Board, Cary, NC, USA |
| Robert Matthew Graham | University of Tennessee Erlanger Oncology & Hematology, Chattanooga, TN, USA | Western IRB, Puyallup, WA, USA |
| Steven McCune | Northwest Georgia Oncology Centers PC, Marietta, GA, USA | Western IRB, Puyallup, WA, USA |
| Primo Lara | UC Davis Comprehensive Cancer Center, Sacramento, CA, USA | UC Davis IRB, Sacramento, CA, USA |
| Neeraj Agarwal, | Huntsman Cancer Institute, Salt Lake City, UT, USA | University of Utah Institutional Review Board, Salt Lake City, UT, USA |
| Michael S. Gordon | Scottsdale Healthcare, Scottsdale, AZ, USA | Western IRB, Puyallup, WA, USA |
| Ruth McGinn/Ray McDermott | Adelaide & Meath Hospital, Dublin, Ireland | Clinical Research Ethics Committee – University College Cork, Cork, Ireland |
| Emmet Jordan | University Hospital Waterford, Waterford, lreland | Clinical Research Ethics Committee – University College Cork, Cork, Ireland |
| Daniel Heinrich | Akershus University Hospital, Nordbyhagen, Norway | REK soer-oest D, Oslo, Norway |
| Ase Haug | Helse Bergen HF Haukeland, Bergen, Norway | REK soer-oest D, Oslo, Norway |
| Heidi Knobel | St Olavs Hospital, Trondheim, Norway | REK soer-oest D, Oslo, Norway |
| Sara Gunnestad Ribe | Sorlandet sykehus HF, Kristiansand, Norway | REK soer-oest D, Oslo, Norway |
| Oyvind Krohn Tennoe | Sykehuset Oestfold, Gralum Norway | REK soer-oest D, Oslo, Norway |
| Hege Sagstuen Haugnes | Universitetssykehuset I Nord Norge, Tromso, Norway | REK soer-oest D, Oslo, Norway |
| Anne Berit Rodal Erdal/Carl W Langberg | Oslo universitetssykehus, Oslo, Norway | REK soer-oest D, Oslo, Norway |
| Alejo Rodriguez-Vida | Hospital del Mar, Barcelona, Spain | CEIC Autonomico de Galicia (SERGAS), Santiago de Compostela, Spain |
| Olatz Etxaniz | Institut Catala d Oncologia Hospital Germans Trias i Pujol, Barcelona, Spain | CEIC Autonomico de Galicia (SERGAS), Santiago de Compostela, Spain |
| Enrique Gallardo Diaz | Hospital Parc Tauli, Sabadell, Spain | CEIC Autonomico de Galicia (SERGAS), Santiago de Compostela, Spain |
| Maria Jose Juan Fita | Instituto Valenciano de Oncologia, Valencia, Spain | CEIC Autonomico de Galicia (SERGAS), Santiago de Compostela, Spain |
| Urbano Anido Herranz | Hospital Clinico Universitario de Santiago, Santiago de Compostela, Spain | CEIC Autonomico de Galicia (SERGAS), Santiago de Compostela, Spain |
| Jose Angel Arranz | Hospital General Universitario Gregorio Maranon, Madrid, Spain | CEIC Autonomico de Galicia (SERGAS), Santiago de Compostela, Spain |
| Jesus Garcia-Donas Jimenez | Hospital Universitario HM Sanchinarro, Madrid, Spain | CEIC Autonomico de Galicia (SERGAS), Santiago de Compostela, Spain |
| James Larkin | The Royal Marsden Foundation Trust, London, UK | Fulham Research Ethics Committee, Manchester, UK |
| Lajos Geczi | Orszagos Onkologiai Intezet, Budapest, Hungary | Egeszsegugyi Tudomanyos Tanacs, Budapest, Hungary |
| Janos Revesz | Borsod-Abauj-Zemplen Megyei Korhaz es Egyetemi OktatoKorhaz, Miskolc, Hungary | National Institute of Pharmacy, Budapest, Hungary |
| Boris Yakovlevich Alekseev | National Medical Research Radiology Centre, Moscow, Russia | Independent Ethics Committee of the FSBI “Russian Scientific Center of Roentgenoradiology” Ministry of Health of the Russian Federation, Moscow, Russia |
| Rustem Airatovich Gafanov | Russian Scientific Center of Roentgenoradiology, Moscow, Russia | Ethics Committee of the FSBI “Central Clinical Hospital with outpatient Clinic” Administration of the President of the Russian Federation, Moscow, Russia |
| Dmitry Aleksandrovich Nosov | Central Clinical Hospital with outpatient Clinic, Moscow, Russia | Ethics Committee of the FSBI “Central Clinical Hospital with outpatient Clinic” Administration of the President of the Russian Federation, Moscow, Russia |
| Andrey Semenov | Ivanovo regional oncology dispensary, Ivanovo, Russia | Local Ethics Committee of the Regional Budget Health Care Institution Ivanovo regional oncology dispensary, Ivanovo, Russia |
| Vladimir Anatolyevich Kostorov | Leningrad Regional Oncology Center, Saint-Petersburg, Russia | Local Ethics Committee of the FSBI “Leningrad Regional Oncology Center,” Saint-Petersburg, Russia |
| Oleg Nikolaevich Lipatov | Republican Clinical Oncology Dispensary of Republic of Bashkortostan, Bashkortostan, Russia | Ethics Committee of the Republican Clinical Oncology Dispensary of Republic of Bashkortostan – Bashkortostan, Russia |
| Christian Caglevic/ Jose Luis Leal/Luis Villanueva | Fundacion Arturo Lopez Perez FALP, Santiago, Chile | Comite de Etica Cientifico del Servicio de Salud Metropolitano Oriente, Santiago, Chile |
| Alejandro Acevedo | Oncocentro, Valparaiso, Chile | Comite Etico Cientifico Hospital Gustavo Fricke Servicio de Salud Viña del Mar Quillota, Valparaiso, Chile |
| Carolina Ibanez | Pontificia Universidad Catolica de Chile, Santiago, Chile | Comite Etico Cientifico de Ciencias de la Salud UC, Santiago, Chile |
| Valentina Hornig | Clinica Alemana de Osorno, Osorno, Chile | Comite Etico Cientifico Servicio de Salud Valldivia, Valdivia, Chile |
| Howard Paul Gurney | Westmead Hospital, Westmead, Australia | North Sydney Local Health District HREC, St. Leonards, Australia |
| Craig Gedye | Calvary Mater Newcastle, Waratah, Australia | North Sydney Local Health District HREC, St. Leonards, Australia |
| David Pook | Cabrini Health, Melbourne, Australia | North Sydney Local Health District HREC, St. Leonards, Australia |
| Jae Lyun Lee | Asan Medical Center, Seoul, South Korea | Asan Medical Center IRB/IEC, South Korea |
| Minjeong Kim/Sun Young Rha | Severance Hospital Yonsei University Health System, Seoul, South Korea | Severance Hospital Yonsei University Health System IRB/IEC, , Seoul, South Korea |
| Se Hoon Park | Samsung Medical Center, Seoul, South Korea | Samsung Medical Center IRB/EC, Seoul, South Korea |
| Woo Kyun Bae | Chonnam National University Hwasun Hospital, Hwasun gun, South Korea | Chonnam National University Hwasun Hospital IRB, Seoul, South Korea |
| Chia-Chi Lin | National Taiwan University Hospital, Taipei, Taiwan | National Taiwan University Hospital. Ethics Review Committee B, Taipei, Taiwan |
| Tzu-Chun Wei/Tzu-Ping Lin | Taipei Veterans General Hospital, Taipei, Taiwan | Taipei Veterans General Hospital Ethics Review Committee, Taipei, Taiwan |
| See-Tong Pang | Chang Gung Med Foundation – Linkou, Taoyuan, Taiwan | Chang Gung Medical Foundation Institutional Review Board A, Taoyuan, Taiwan |
| Chao-Hsiang Chang | China Medical University Hospital, Taipei, Taiwan | China Medical University Hospital Research Ethics Committee, Taichung, Taiwan |
| Wen-Pin Su | National Cheng Kung University Hospital, Tainan, Taiwan | IRB Chairman: Thy-Sheng Lin, Tainan, Taiwan |
| Yu-Li Su | Chang Gung Med Foundation. Kaohsiung Branch, Kaohsiung, Taiwan | Chang Gung Medical Foundation Institutional Review Board, Taipei, Taiwan |
| Go Kimura | Nippon Medical School Hospital, Tokyo, Japan | Nippon Medical School Hospital Institutional Review Board, Tokyo, Japan |
| Hiroaki Matsumoto | Yamaguchi University Hospital, Ube, Japan | Yamaguchi University Hospital Institutional Review Board, Ube, Japan |
| Ryuichi Mizuno | Keio University Hospital, Tokyo, Japan | Keio University Hospital Institutional Review Board, Ube, Japan |
| Hirotsugu Uemura | Kindai University Hospital, Osaka, Japan | Kindai University Hospital Institutional Review Board, Osaka, Japan |
| Satoshi Anai | Nara Medical University Hospital, Nara, Japan | Nara Medical University Hospital Institutional Review Board, Nara, Japan |
| Naoya Masumori | Sapporo Medical University Hospital, Hokkaido, Japan | Sapporo Medical University Hospital Institutional Review Board, Hokkaido, Japan |
| Mustafa Erman | Hacettepe University Medical Faculty, Ankara, Turkey | Medeniyet Universitesi Klinik Aras Etik Kurulu Hacettepe University Faculty of Medicine Ethics Committee, Ankara, Turkey |
| Tarkan Yetisyigit | Namik Kemal Universitesi Tip Fakultesi, Tekirdas, Turkey | Medeniyet Universitesi Klinik Aras Etik Kurulu Hacettepe University Faculty of Medicine Ethics Committee, Ankara, Turkey |
| Erhan Gokmen | Ege Universitesi Tip Fakultesi  Ege UTF Tulay Aktas Onkoloji, Izmir, Turkey | Medeniyet Universitesi Klinik Aras Etik Kurulu Hacettepe University Faculty of Medicine Ethics Committee, Ankara, Turkey |
| Mert Basaran | Istanbul Universitesi Onkoloji Enstitusu, Istanbul, Turkey | Medeniyet Universitesi Klinik Aras Etik Kurulu Hacettepe University Faculty of Medicine Ethics Committee, Ankara, Turkey |
| Ozgur Ozyilkan | Baskent Universitesi Dr. Turgut Noyan Uygulama ve Arastirma Merkezi, Adana, Turkey | Medeniyet Universitesi Klinik Aras Etik Kurulu Hacettepe University Faculty of Medicine Ethics Committee, Ankara, Turkey |
| Christopher DiSimone | Arizona Oncology Associates PC- HOPE, Tucson, AZ, USA | US Oncology and Research IRB, The Woodlands, TX, USA |
| John T Fitzharris | Willamette Valley Cancer Institute and Research Center, Eugene, OR, USA | US Oncology and Research IRB, The Woodlands, TX, USA |
| Rami Owera | Woodlands Medical Specialists, PA, Pensacola, FL, USA | US Oncology and Research IRB, The Woodlands, TX, USA |
| William Houck,III | Shenandoah Oncology, P.C., Winchester, VA, USA | US Oncology and Research IRB, The Woodlands, TX, USA |
| Mark D. Kochenderfer | Oncology & Hematology Associates of Southwest Virginia, Inc., DBA Blue Ridge Cancer Care, Roanoke, VA, USA | US Oncology and Research IRB, The Woodlands, TX, USA |
| David R. Shaffer | New York Oncology Hematology P.C, Albany NJ, USA | US Oncology and Research, The Woodlands, TX, USA |

**Supplemental Figure 1.** Box and whisker plot showing change from baseline to week 3 in serum kynurenine among patients in the both the pembrolizumab plus epacadostat arm and the sunitinib/pazopanib arm. Kynurenine levels at C1D1 and C2D1 were compared using paired t-tests within each treatment arm The dotted line indicates median kynurenine levels in healthy subjects (1.5 μM)[1] *Epa* , epacadostat, *Pembro* pembrolizumab, *SoC* standard of care


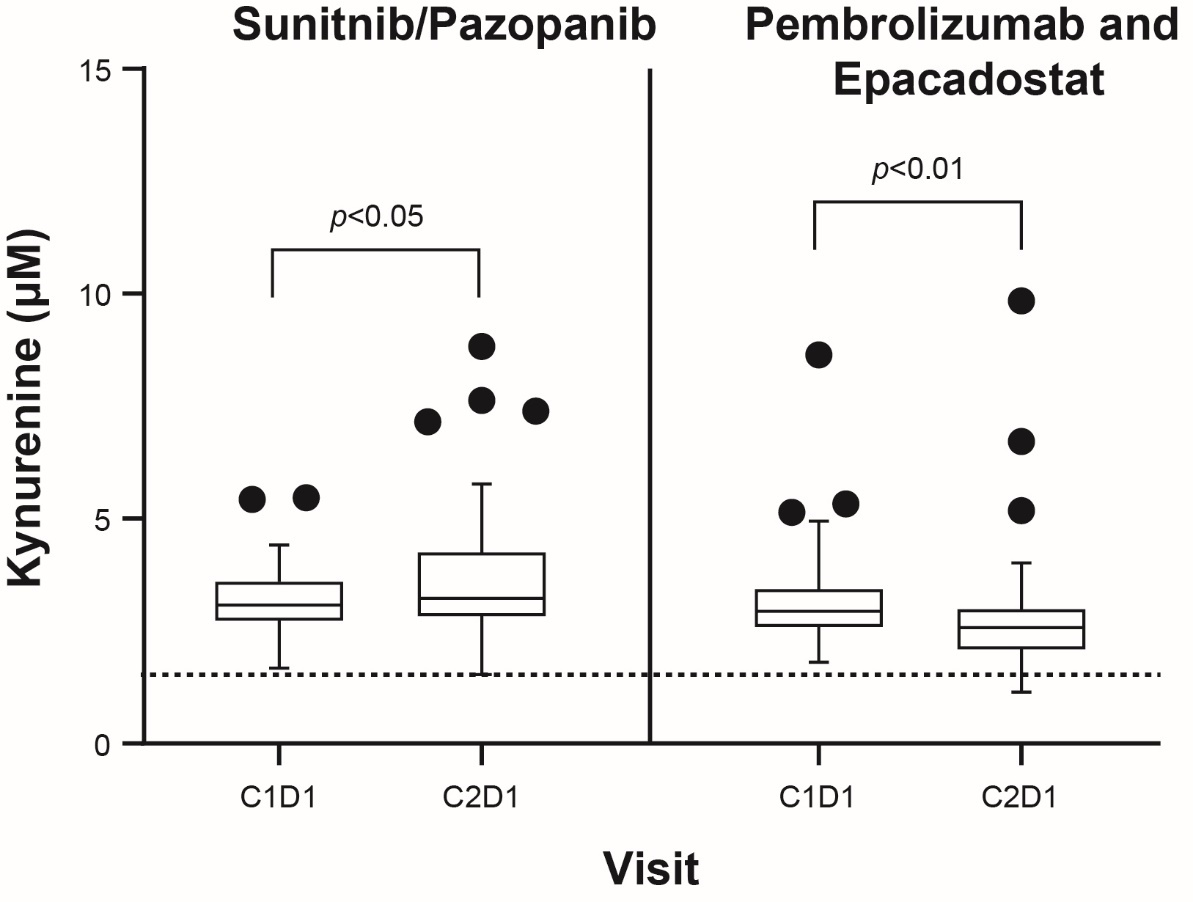


**Reference**

1. Beatty GL, O'Dwyer PJ, Clark J, Shi JG, Bowman KJ, Scherle PA, et al. First-in-human phase I study of the oral inhibitor of indoleamine 2,3-dioxygenase-1 epacadostat (INCB024360) in patients with advanced solid malignancies. Clin Cancer Res. 2017;23(13):3269–76.
